# Discovery of dachshund 2 protein as a novel biomarker of poor prognosis in epithelial ovarian cancer

**DOI:** 10.1186/1757-2215-5-6

**Published:** 2012-01-27

**Authors:** Björn Nodin, Marie Fridberg, Mathias Uhlén, Karin Jirström

**Affiliations:** 1 Department of Clinical Sciences, Division of Pathology, Lund University, Skåne University Hospital, 221 85 Lund, Sweden; 2 Department of Proteomics, AlbaNova University Center, Royal Institute of Technology, 106 91 Stockholm, Sweden; 3 Science for Life Laboratory, Royal Institute of Technology, 106 91 Stockholm, Sweden

**Keywords:** DACH2, ovarian cancer, prognosis

## Abstract

**Background:**

The Dachshund homolog 2 (*DACH2*) gene has been implicated in development of the female genital tract in mouse models and premature ovarian failure syndrome, but to date, its expression in human normal and cancerous tissue remains unexplored. Using the Human Protein Atlas as a tool for cancer biomarker discovery, DACH2 protein was found to be differentially expressed in epithelial ovarian cancer (EOC). Here, the expression and prognostic significance of DACH2 was further evaluated in ovarian cancer cell lines and human EOC samples.

**Methods:**

Immunohistochemical expression of DACH2 was examined in tissue microarrays with 143 incident EOC cases from two prospective, population-based cohorts, including a subset of benign-appearing fallopian tubes (n = 32). A nuclear score (NS), i.e. multiplier of staining fraction and intensity, was calculated. For survival analyses, cases were dichotomized into low (NS < = 3) and high (NS > 3) using classification and regression tree analysis. Kaplan Meier analysis and Cox proportional hazards modelling were used to assess the impact of DACH2 expression on survival. DACH2 expression was analysed in the cisplatin sensitive ovarian cancer cell line A2780 and its cisplatin resistant derivative A2780-Cp70. The specificity of the DACH2 antibody was tested using siRNA-mediated silencing of DACH2 in A2780-Cp70 cells.

**Results:**

DACH2 expression was considerably higher in the cisplatin resistant A2780-Cp70 cells compared to the cisplatin-sensitive A2780 cells. While present in all sampled fallopian tubes, DACH2 expression ranged from negative to strong in EOC. In EOC, DACH2 expression correlated with several proteins involved in DNA integrity and repair, and proliferation. DACH2 expression was significantly higher in carcinoma of the serous subtype compared to non-serous carcinoma. In the full cohort, high DACH2 expression was significantly associated with poor prognosis in univariable analysis, and in carcinoma of the serous subtype, DACH2 remained an independent factor of poor prognosis.

**Conclusions:**

This study provides a first demonstration of DACH2 protein being expressed in human fallopian tubes and EOC, with the highest expression in serous carcinoma where DACH2 was found to be an independent biomarker of poor prognosis. Future research should expand on the role of DACH2 in ovarian carcinogenesis and chemotherapy resistance.

## Background

Epithelial ovarian cancer (EOC) is the fifth most common cause of cancer-related death in women and the leading cause of death from gynaecological malignancy [1]. Etiological factors involved in ovarian carcinogenesis remain poorly defined, and effective treatment protocols are limited. The poor ratio of survival to incidence is related to the high percentage of cases diagnosed at an advanced stage, and the symptoms of EOC are often vague and overlap with other more common gastrointestinal and gynaecological diseases. Despite aggressive surgery and chemotherapy, most patients relapse within 3 to 5 years, and the median time to relapse is 15 months after diagnosis [2]. Thus, there is an urgent need for the identification of novel diagnostic, prognostic, and predictive biomarkers for development of personalized therapeutic regimens for ovarian cancer patients.

Using the Human Protein Atlas http://www.proteinatlas.org as a tool for antibody based biomarker discovery [3,4], the Dachshund 2 (DACH2) protein was identified as being differentially expressed among EOC samples, ranging from negative to strong nuclear staining. Based on this observation, we hypothesized that DACH2 might be involved in ovarian carcinogenesis and, hence, a putative prognostic and treatment predictive biomarker in EOC.

The *dachshund (DACH) *gene was first described in Drosophila, where it encodes a nuclear protein involved in development of the eyes, limbs and genital disc [5,6]. While Drosophila has a single *dachshund *gene, two *DACH *genes, *DACH1 *and *DACH2*, have been found in mice, humans and chicken [7-10] In mice, the *DACH1 *and *DACH2 *genes show functional redundancy during development of the female genital tract, whereby defects are associated with Müllerian but not Wolffian duct development [11]. In humans, the *DACH2 *gene has been implicated in premature ovarian failure (POF) syndrome [12,13], indicating that alterations of the human DACH2 protein may constitute a risk-factor for POF by altering the correct process of ovarian follicle differentiation [13].

While the role of DACH2 in human tumourigenesis remains unexplored, alterations of DACH1 expression has been described in several cancer forms, e.g. breast [14], prostate [15], endometrial [16], gastric [17] and ovarian cancer [18]. The prognostic value of DACH1 seems to be cancer-type dependent in that reduced DACH1 levels have been associated with poor prognosis in breast, gastric, and endometrial cancer [16,17,19] and with tumour progression in prostate cancer [15], whilst in EOC, DACH1 has been shown to be up-regulated in advance-stage ovarian cancer and promote resistance to TGF-β signaling [18].

The aim of this study was to investigate the prognostic role of DACH2 protein expression in ovarian cancer, by immunohistochemical analysis of 154 EOC samples from two prospective, population-based cohorts. DACH2 levels were also assessed in a cisplatin sensitive and resistant ovarian cancer cell line, respectively.

## Methods

### Patients

The study cohort is a merge of all incident cases of epithelial ovarian cancers in the population-based prospective cohort studies Malmö Diet and Cancer Study (n = 101)[20] and Malmö Preventive Medicine Cohort (n = 108)[21] until Dec 31st 2007. Thirty-five patients participated in both studies, and archival tumour tissue could be retrieved from 154 of the total number of 174 cases. Cases were identified from the Swedish Cancer Registry up until 31 Dec 2006, and from The Southern Swedish Regional Tumour Registry for the period of 1 Jan - 31 Dec 2007. All tumours were re-evaluated regarding histological subtype and histological grade by a board certified pathologist (KJ). Information regarding clinical stage was obtained from the medical charts, following the standardized FIGO classification of tumour staging. Information on residual tumour after surgery was not available. Standard adjuvant therapy was platinum-based chemotherapy, from the 1990s given in combination with paclitaxel.

Histopathological, clinical and treatment data were obtained from the clinical- and/or pathology records. Information on vital status and cause of death was obtained from the Swedish Cause of Death Registry up until 31 Dec 2008. Follow-up started at date of diagnosis and ended at death, emigration or 31 Dec 2008, whichever came first. After a median follow-up of 2.65 years (range 0-21), 105 patients (68.2%) were dead and 49 (31.8%) alive. Patient-and tumour characteristics of the cohort have been described in detail previously [22-24]. Ethical permissions for the MDCS (Ref. 51/90), and the present study (Ref. 530/2008), were obtained from the Ethics Committee at Lund University.

### Tissue microarray construction and immunohistochemistry

Areas representative of cancer were marked on full-face haematoxylin and eosin stained sections and TMAs constructed as previously described [25]. In brief, 2-4 1.0 mm cores were taken from each tumour and mounted in a new recipient block using a semi-automated arraying device (TMArrayer; Pathology Devices, Inc, Westminster, MD, USA).

For immunohistochemical analysis of DACH2, 4 μm TMA-sections were automatically pretreated using the PT-link system (DAKO, Glostrup, Denmark) and then stained in a Autostainer Plus (DAKO) with a polyclonal anti-DACH2 antibody (HPA0000258, Atlas Antibodies AB, Stockholm, Sweden) diluted 1:50. Immunohistochemistry for RBM3, Chek1, Chek2, MCM3, estrogen receptor α (ER), progesterone receptor (PR), and androgen receptor (AR) was performed as previously described [22-24]. Ki67 was analysed using a monoclonal antibody (MIB-1, DAKO, diluted 1:200)

### Analysis of immunohistochemical staining

DACH2 was primarily expressed in the nucleus and for assessment of DACH2 expression, both the fraction of positive cells and staining intensity were taken into account. Nuclear fraction was categorized into four groups, namely 0 (0-1%), 1 (2-25%), 2 (26-75) and 3 (> 75%) and nuclear staining intensity denoted as 0-3, whereby 0 = negative, 1 = intermediate, 2 = moderate and 3 = strong intensity. A combined nuclear score (NS) was then constructed as a multipler of DACH2 nuclear fraction and intensity, thus ranging from 0 to 9. Ki67 was annotated as the fraction of positive staining cells and denoted as 0 (0-1%), 1(2-25%), 2(26-50%) and 3(> 50%).

### Cell lines and reagents

The human ovarian cancer cell line A2780 and the cisplatin-resistant variant A2780-Cp70 were maintained in RPMI-1640 supplemented with glutamine, 10% fetal bovine serum and 1% pencillin/streptomycin in a humidified incubator of 5% CO2 at 37°C.

### Real-time quantitative PCR and Western Blotting

Total RNA isolation (RNeasy, QIAgen, Hilden, Germany), cDNA synthesis (Reverse Transcriptase kit, Life Technologies, Carlsbad, Ca, USA) and quantitative real-time PCR (qRT-PCR) analysis of DACH2 expression with TaqMan Gene Expression Assay (Hs 00364968, Life Technologies) was performed according to the manufacturers instructions. Quantification of expression levels were calculated by using the comparative Ct method, normalization according to the house keeping gene 18S (s03928990 g1 RN 18S1; Life Technologies).

For immunoblotting, cells were lysed in ice-cold RIPA buffer (Cayman Chemical Company, Ann Arbor, MI, USA) and supplemented with protease inhibitor cocktail Complete Mini (Roche, Basel, Switzerland). Thirty μg of protein were separated on 4-12% Nu-PAGE Bis-Tris gels and transferred onto iBlot Gel Transfer Stacks Nitrocellulose (Life technologies). DACH2 was detected by the polyclonal DACH2 antibody (HPA 0000258, Atlas Antibodies AB) diluted 1:250 in blocking solution, (WesternBreeze Chemiluminescent Immunodetection System (Life technologies) followed by a secondary antibody solution, Alk-Phos Conjugated, Anti-Rabbit (WesternBreeze Chemiluminescent Immunodetection System, Life technologies) and visualized using WesternBreeze Chemiluminescent Immunodetection System (Life technologies). Membranes were stripped and re-probed with an anti-β-actin antibody (Santa Cruz, Biotechnology, Santa Cruz, CA, USA) at a dilution of 1:1000, to provide a loading control.

### Cell pellet arrays

Cell lines were fixed in 4% formalin and processed in gradient alcohols. Cell pellets were cleared in xylene and washed multiple times in molten paraffin. Once processed, cell lines were arrayed in duplicate 1.0 mm cores using a manual tissue arrayer (Beecher Inc, WI, USA) and IHC was performed on 4 μm sections using the DACH2 antibody diluted 1:50.

### siRNA mediated knockdown of DACH2 gene expression

Transfection with siRNA against DACH2 (Life Technologies) or control siRNA (Life Technologies) was performed with Lipofectamine 2000 (Life Technologies) with a final concentration of 50 nM siRNA. Two independent RNA oligonucleotides (s229511 and s229512, Life Technologies) targeting DACH2 were used.

### Statistical analysis

Spearman's Rho test was used for comparison of DACH2 expression and clinicopathological and tumour biological characteristics. Classification regression tree (CRT) analysis was used to decide optimal cutoff for survival analysis. Kaplan-Meier analysis and log rank test were used to illustrate differences in ovarian cancer specific survival (OCSS) and overall survival (OS) according to DACH2 expression. Cox regression proportional hazards models were used for estimation of hazard ratios (HRs) for death from ovarian cancer or overall causes according to DACH2 expression in both uni- and multivariable analysis, adjusted for stage and differentiation grade. Experimental data are expressed as mean ± SD of three independent experiments. Statistical significance of differences between means was determined by Student's t test. All calculations were performed using IBM SPSS Statistics Version 20 (SPSS Inc, Chicago, IL). All statistical tests were two-sided and a p value < 0.05 was considered statistically significant.

## Results

### Antibody validation and comparison of DACH2 levels in cisplatin-sensitive vs cisplatin-resistant ovarian cancer cells

**Figure 1:**
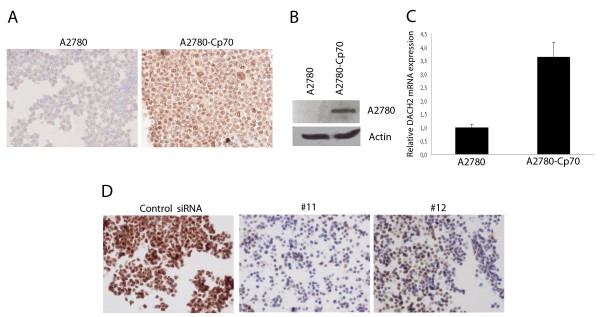
**Expression of DACH2 in the cisplatin-sensitive A2780 ovarian cancer cell line compared to the cisplatin-resistant cell line A2780-Cp70 and validation of the specificity of the DACH2 antibody in A2780 ovarian cancer cells**. Substantially higher DACH2 protein expression was seen in the cisplatin-resistant A2780-Cp70 cell line compared to its parental cisplatin-sensitive A2780 cell line by A) immunocytochemical staining and (B) immunoblotting, showing a major band at 62 kDa corresponding to the expected molecular weight of the DACH2 protein. (C) Relative mRNA expression was also higher in the A2780-Cp70 cells compared to A2780 cells as shown by qRT-PCR analysis. Data shown are mean ± SD of a representative experiment of two independent experiments performed in triplicate. (D) DACH2 protein expression was significantly decreased after transfection with siRNA against DACH2 in A2780-Cp70 cells as shown by immunocytochemistry 72 hrs post-transfection.

DACH2 protein expression, assessed by both IHC and Western blotting, was substantially higher in the cisplatin-resistant derivative A2780-Cp70 cells compared to the parental A2780 cells, in which the DACH2 protein was barely detectable (Figure 1A and 1B). Real-time quantitative PCR (qRT-PCR) confirmed a similar difference whereby there was a 3.7-fold higher level of DACH2 mRNA in the A2780-Cp70 compared to the A2780 cell line (Figure 1C). Cisplatin resistance in the A2780-Cp70 cells relative to the A2780 cells has been confirmed previously [23]. The specificity of the DACH2 antibody was confirmed by siRNA-mediated knockdown of DACH2 in A2780/Cp70 cells. IHC performed on formalin fixed, paraffin embedded siRNA transfected A2780/Cp70 cells revealed a marked decrease in immunoreactivity in the DACH2 knockdown cells compared to controls as visualized by IHC on cell pellets (Figure 1D).

### Immunohistochemical expression of DACH2 in fallopian tubes and EOC

**Figure 2:**
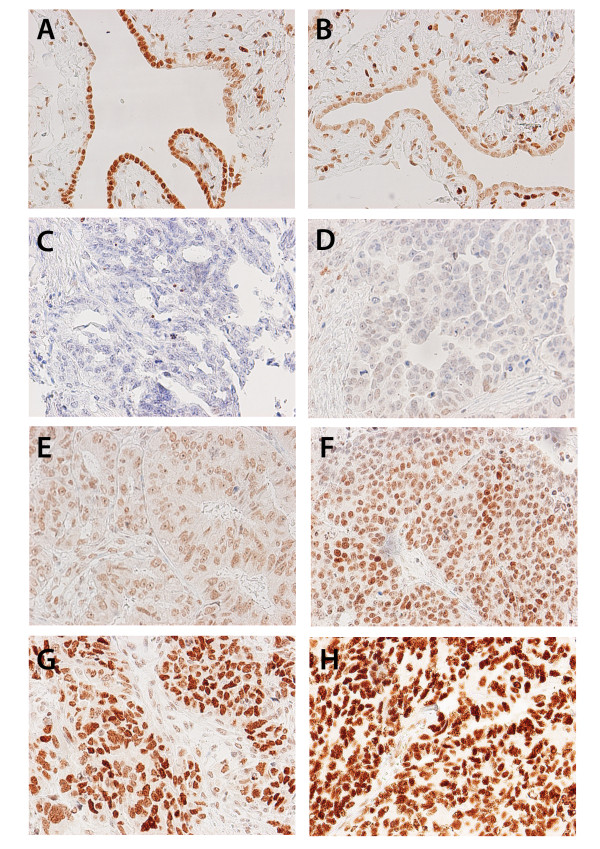
**Immunohistochemical images of DACH2 staining in fallopian tubes and ovarian cancer**. Images (20× magnification) representing immunohistochemical expression of DACH2 in (A, B) fallopian tubes, and EOC ranging from (C) negative, (D) weak intensity in few cells, (E) weak intensity in majority of cells, (F) moderate to strong intensity in majority of cells, (G) strong intensity in majority of cells and (H) strong intensity in all tumour cells.

**Figure 3:**
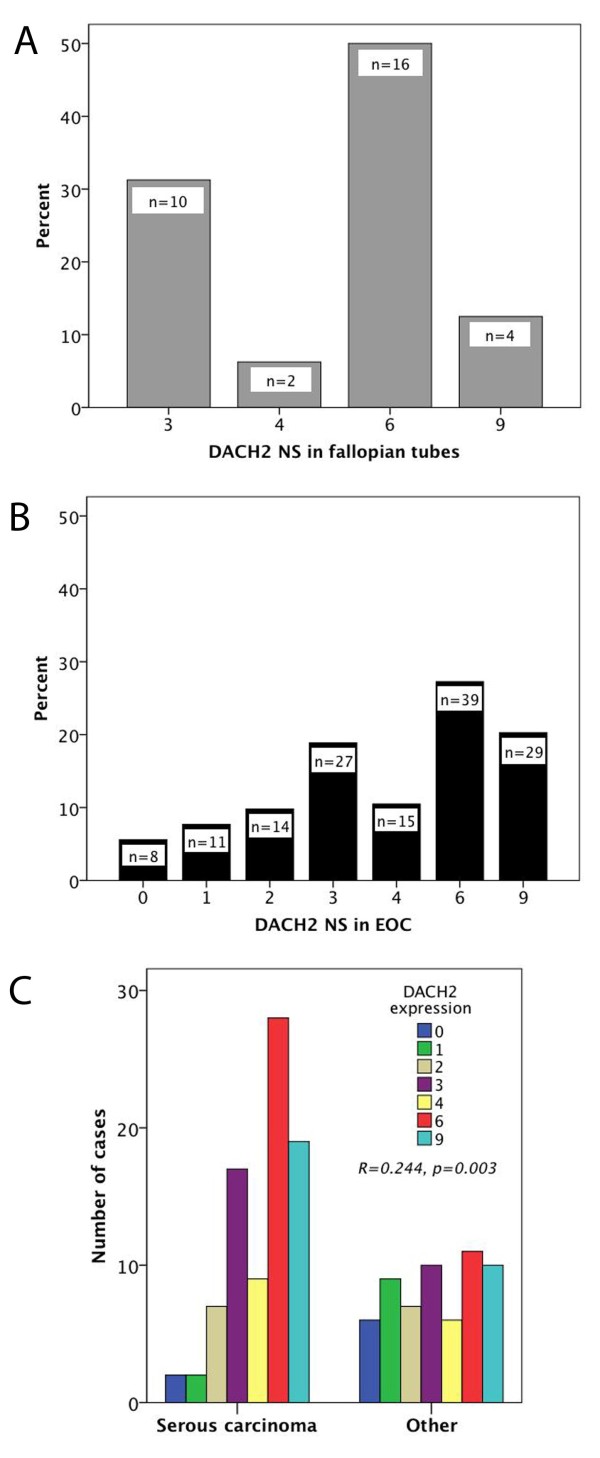
**Distribution of DACH2 expression in fallopian tubes and ovarian cancer**. Bar charts visualizing the staining distribution of DACH2 in (A) fallopian tubes and (B) ovarian cancer, and (C) in serous vs non-serous carcinoma. NS = nuclear score, e.g. a multiplier of fraction (0-3) and intensity (0-3) of staining.

Following antibody optimisation and staining, DACH2 expression could be evaluated in 32/38 (84.2%) samples from fallopian tubes and 143/154 (92.9%) EOC cases. There was no obvious heterogeneity in DACH2 expression between duplicate TMA cores. Images representing different patterns of expression in tubal epithelium and EOC are shown in Figure 2 A-H, whereby A-B represent tubal epithelium, C-E tumours with a NS < = 3 and F-H tumours with a NS > 3. As regards the staining distribution, expression of DACH2 protein was evident in all fallopian tubes with nuclear scores ranging from 3-9 (Figure 3A). A wider range of DACH2 expression was observed in EOC, where 8 (5.6%) cases were denoted as DACH2 negative and 33 (23.1%) cases had a NS < 3, e.g. lower than in the tubal epithelium (Figure 3B). There was however no statistically significant difference in DACH2 expression in tubal epithelium and EOC in cases from which paired samples had been analysed (n = 30), of whom 2 had DACH2 negative tumours, 5 had tumours with a NS < 3, and the remaining cases (n = 25) had a NS > = 3 in the invasive component (data not shown). There was no significant difference in DACH2 expression between cancer located to the ovaries and metastatic deposits (data not shown). DACH2 staining was significantly higher in carcinomas of the serous subtype compared to nonserous carcinomas (R = 0.244, p = 0.003) (Figure 3C). Comparison of different histological subtypes within non-serous carcinomas, i.e. mucinous, endometroid and clear cell carcinomas revealed no significant difference in the distribution of DACH2 staining (data not shown). The distribution of DACH2 in tubal epithelium was similar in serous and non-serous carcinomas (data not shown).

### Association between DACH2 expression, clinicopathological characteristics and markers of proliferation and DNA integrity

Next, we examined the relationship between DACH2 expression (NS) and established clinicopathological and investigative parameters (Table 1). In the full cohort, DACH2 expression showed a positive correlation to Ki67, Chk1, Chk2 and MCM3 expression. There was no significant correlation between DACH2 expression and established clinicopathological factors, i.e. clinical stage and grade, nor to RBM3, AR, ER or PR expression. In the serous subtype, DACH2 was not significantly associated with any other clinicopathological or tumour biological parameters (Table 1).

**Table 1:** Associations between DACH2 expression and clinicopathological parameters in all patients and patients with serous carcinoma.

	All	Serous carcinoma
Factor	DACH2	DACH2
**Age**		
R	0.044	0.078
p	0.603	0.483
n	143	84

**Differentiation grade**		
R	-0,031	-0.189
p	0.716	0.086
n	143	84

**Clinical stage**		
R	0.025	0.078
p	0.779	0.494
n	131	80

**Ki67**		
R	0.208	0.086
p	0.013*	0.435
n	141	84

**AR**		
R	0.028	-0.045
p	0.738	0.683
n	143	84

**ER**		
R	0.122	-0.067
p	0.151	0.554
n	139	81

**PR**		
R	0.130	0.085
p	0.126	0.437
n	141	85

**RBM3**		
R	-0,072	-0.167
p	0.393	0.129
n	141	84

**Chek1**		
R	0.194	0.139
p	0.024*	0.225
n	134	78

**Chek2**		
R	0.182	0.155
p	0.032*	0.164
n	139	78

**MCM3**		
R	0.252	0.104
p	0.003**	0.360
n	134	79

R = Spearman's correlation coefficient, p = p-value, n = number of cases available for analysis. ER = estrogen receptor, PR = progesterone receptor, AR = Androgen receptor. *significance at 5% level, ** significance at 1% level. The analysis are based on multipliers of staining intensity and fraction (nuclear score) for DACH2, RBM3, Chek1, Chek2 and MCM3 and categories of nuclear fraction for Ki67, AR, ER, and PR.

### Association between DACH2 expression and survival from EOC

**Figure 4:**
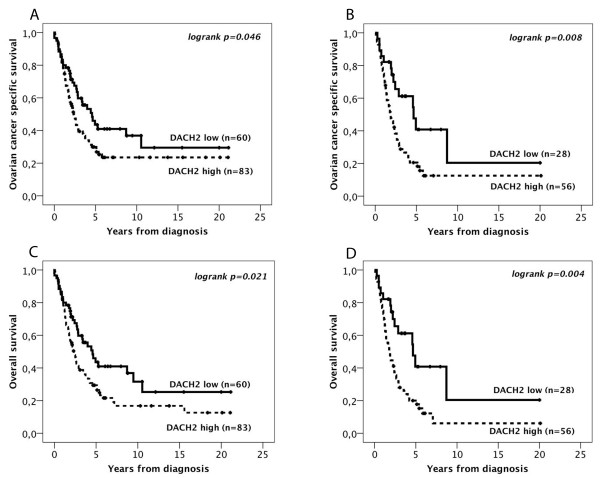
**Kaplan-Meier estimates of ovarian cancer specific and overall survival in all patients according to DACH2 expression**. Kaplan Meier analysis of ovarian cancer specific and overall survival in strata of low and high DACH2 expression in (A, C) all patients, and (B, D) serous carcinoma. The categories of staining were determined according to the nuclear score (NS), e.g. a multiplier of fraction and intensity, whereby low expression = NS < = 3 and high expression = NS > 3.

CRT analysis suggested an optimal cutoff point at NS > 3 to determine the impact of DACH2 expression on OCSS and OS. Kaplan Meier analysis of the entire cohort (n = 143) demonstrated a significantly reduced OCSS (p = 0.046) and OS (p = 0.021) for tumours expressing high levels of DACH2 (Figure 4A, B). These associations were accentuated in the subgroup of serous carcinoma (n = 84) for both OCSS (p = 0.008) and OS (p = 0.004) (Figure 4C, D). The associations between DACH2 expression and survival were confirmed in univariable Cox regression analysis (Table 2). In multivariable analysis, DACH2 remained an independent prognostic factor in patients with serous carcinoma for both OCSS (HR = 2.01, 95% CI 1.05-3.85, p = 0.035) and OS (HR = 2.13, 95% CI 1.12-4.08, p = 0.022), but not in the full cohort (Table 2). DACH2 was not prognostic in separate analysis of other histological subgroups (data not shown). Ki67 expression was not prognostic, neither in the full cohort nor in the subgroup of serous carcinoma (data not shown).

**Table 2:** Relative risks of death from ovarian cancer and overall death according to DACH2 expression in all patients and patients with serous carcinoma.

	Ovarian cancer specific survival			Overall survival		
	
	HR(95%CI)	*p-value*	*n(events)*	HR(95%CI)	*p-value*	*n(events)*
**All**		*Univariable*			*Univariable*	
DACH2 low	1,00	0.048	60	1,00	0.022	60
DACH2 high	1.54(1.00-2.35)		83	1.63(1.07-2.47)		83
		*Multivariable*			*Multivariable*	
DACH2 low	1,00	0.182	55	1,00	*0.088*	55
DACH2 high	1.36(0.87-2.12)		76	1.57(0.95-2.28)		76

**Serous carcinoma**		*Univariable*			*Univariable*	
DACH2 low	1,00	*0.010*	28	1,00	*0.005*	28
DACH2 high	2.21(1.21-4.04)		56	2.34(1.23-4.26)		56
		*Multivariable*			*Multivariable*	
DACH2 low	1,00	*0.035*	*25*	1,00	*0.022*	25
DACH2 high	2.01(1.05-3.85)		51	2.13(1.12-4.08)		51

Cox uni- and multivariable analysis of relative risks of death from ovarian cancer and overall death according to DACH2 expression in all patients and patients with serous carcinoma. HR = Hazard ratio. The categories of staining were determined according to the nuclear score (NS), e.g. a multiplier of fraction and intensity, whereby low expression = NS < = 3 and high expression = NS > 3. Multivariate analysis included adjustment for differentiation grade (low-intermediate vs high) and clinical stage (1-2 vs 3 and 4).

## Discussion

The results from this study provide a first demonstration of DACH2 being abundantly expressed at the protein level in human fallopian tubes and EOC. Moreover, DACH2 expression was found to be significantly higher in EOC of the serous subtype compared to non-serous carcinoma, and an independent predictor of poor survival in the former.

In the full cohort of EOC, there was a positive correlation between expression of DACH2 and crucial checkpoint proteins and regulators of cellular DNA damage response Chek1 and Chek2 [26], as well as MCM3, a key component of the DNA replication licensing system [27]. High expression of Chek1, Chek2 and MCM3 has previously been demonstrated to be associated with a poor prognosis in the here studied cohort of tumours, although not independent of other established clinicopathological parameters [24]. Moreover, the positive association between DACH2 and Ki67 further supports a role for DACH2 in conferring a more malignant phenotype in EOC.

The association of DACH2 expression with proteins involved in maintenance of DNA integrity might suggest a role for DACH2 in chemotherapy resistance, a notion further supported by the finding of substantially higher DACH2 expression levels in the cisplatin resistant A2780-Cp70 compared to cisplatin sensitive A2780 ovarian cancer cells. It would therefore be of interest to address the molecular basis for how DACH2 might modulate the effects of both platinum and taxane-based chemotherapy in future mechanistic studies. However, the association between DACH2 expression and other investigative markers, e.g. Chek1, Chek2, MCM3 and Ki67, was only evident in the full cohort and not in the subgroup of serous carcinoma, where DACH2 expression was significantly higher than in non-serous carcinomas, and an independent factor of poor prognosis. These findings, together with the various important developmental functions demonstrated for DACH proteins, not least related to the female genital tract [11-13], indicate that DACH2 might play a more important role in EOC development than in chemotherapy resistance. As DACH2 was found to be expressed in the epithelium of all concomitantly sampled benign-appearing fallopian tubes and a significant proportion of serous carcinomas have been suggested to arise within the fimbrial tubal epithelium [28-30], these observations could indicate differential roles of DACH2 in the progression of serous and non-serous carcinomas, respectively.

While DACH1 has been demonstrated to co-localize with ER in breast cancer and AR in normal prostate and exert repressive effects on both ER and AR mediated signaling [14,15], no correlation was found between expression of DACH2 and AR, ER or PR in the here examined EOC cohort. However, these findings do not exclude a role for DACH2 as a mediator of endocrine signaling in EOC.

Apart from providing a first description of the expression and prognostic significance of DACH2 in EOC, this is also, to our knowledge, the first report of DACH2 expression in any human cancer form. This illustrates the utility of the Human Protein Atlas as a tool for antibody-based biomarker discovery [3], not least in light of the lack of well-validated antibodies in translational research, but also since it facilitates the selection of hypotheses relevant to human disease. The specificity of the polyclonal antibody generated against DACH2 within the HPA project was here further validated by a marked reduction of DACH2 expression in formalin-fixed, paraffin-embedded siDACH2 treated EOC cells compared to controls, confirming its suitability for use in immunohistochemical biomarker studies. Although being a semi-quantitative method, immunohistochemistry has several advantages compared to other assays, not least in the clinical setting, as it is simple to perform, fast and comparatively cheap. More importantly, it allows for marker analysis in different subcellular locations, which might be of crucial importance for prognostication and treatment stratification of patients.

Interestingly, loss of DACH1 expression has been associated with poor prognosis in all hitherto investigated cancer forms with the exception of ovarian cancer, where gene expression profiling analysis identified DACH1 to be up-regulated in advance-stage ovarian cancer and to inhibit TGF-β signaling in ovarian cancer cells [18]. Whether DACH2 is prognostic in other cancer forms, and to what extent this might be cancer-type specific, will be of interest to determine in future studies.

## Conclusions

Using an antibody-based biomarker discovery approach, DACH2 has been identified as a novel biomarker of poor prognosis in EOC. Future studies are warranted to confirm these findings in additional patient cohorts and to further elucidate the role of DACH2 in ovarian carcinogenesis, progression and chemotherapy response.

## List of abbreviations

DACH2: Dachshund2; EOC: Epithelial ovarian cancer; NS:Nuclear score; OCSS: Ovarian cancer specific survival; OS: Overall survival; AR: Androgen receptor; ER: Estrogen receptor α; PR: Progesterone receptor.

## Competing interests

A patent application has been filed related to the use of DACH2 as a prognostic and treatment predictive biomarker in EOC.

## Authors' contributions

BN performed statistical analysis, carried out the experimental studies and drafted the manuscript. MF assisted with the experimental studies and helped to draft the manuscript, MU participated in the design of the study and technical assistance. KJ conceived of the study and participated in its design and coordination and helped to draft the manuscript. All authors read and approved the final manuscript.
